# Blood-based biological ageing and red cell distribution width are associated with prevalent Parkinson’s disease: findings from a large Italian population cohort

**DOI:** 10.3389/fendo.2024.1376545

**Published:** 2024-04-10

**Authors:** Alessandro Gialluisi, Alfonsina Tirozzi, Simona Costanzo, Maria Ilenia De Bartolo, Daniele Belvisi, Sara Magnacca, Amalia De Curtis, Stefania Falciglia, Moreno Ricci, Chiara Cerletti, Maria Benedetta Donati, Alfredo Berardelli, Giovanni de Gaetano, Licia Iacoviello

**Affiliations:** ^1^ Department of Epidemiology and Prevention, IRCCS NEUROMED, Pozzilli, Italy; ^2^ Department of Medicine and Surgery, LUM University, Casamassima, Italy; ^3^ IRCCS NEUROMED, Pozzilli, Italy; ^4^ Department of Human Neurosciences, Sapienza University of Rome, Rome, Italy; ^5^ UOC Governance del Farmaco, Azienda Sanitaria Regionale del Molise –ASREM, Campobasso, Italy

**Keywords:** Parkinson’s disease, PhenoAge, biological aging, red cell distribution width, inflammation, oxidative stress

## Abstract

**Background:**

Aging clocks tag the actual underlying age of an organism and its discrepancy with chronological age and have been reported to predict incident disease risk in the general population. However, the relationship with neurodegenerative risk and in particular with Parkinson’s Disease (PD) remains unclear, with few discordant findings reporting associations with both incident and prevalent PD risk.

**Objective:**

To clarify this relationship, we computed a common aging clock based on blood markers and tested the resulting discrepancy with chronological age (ΔPhenoAge) for association with both incident and prevalent PD risk.

**Methods:**

In a large Italian population cohort - the Moli-sani study (N=23,437; age ≥ 35 years; 52% women) - we carried out both Cox Proportional Hazards regressions modelling ΔPhenoAge as exposure and incident PD as outcome, and linear models testing prevalent PD as exposure and ΔPhenoAge as outcome. All models were incrementally adjusted for age, sex, education level completed and other risk/protective factors previously associated with PD risk in the same cohort (prevalent dysthyroidism, hypertension, diabetes, use of oral contraceptives, exposure to paints, daily coffee intake and cigarette smoking).

**Results:**

No significant association between incident PD risk (209 cases, median (IQR) follow-up time 11.19 (2.03) years) and PhenoAging was observed (Hazard Ratio [95% Confidence Interval] = 0.98 [0.71; 1.37]). However, a small but significant increase of ΔPhenoAge was observed in prevalent PD cases vs healthy subjects (β (Standard Error) = 1.39 (0.70)). An analysis of each component biomarker of PhenoAge revealed a significant positive association of prevalent PD status with red cell distribution width (RDW; β (SE) = 0.46 (0.18)). All the remaining markers did not show any significant evidence of association.

**Conclusion:**

The reported evidence highlights systemic effects of prevalent PD status on biological aging and red cell distribution width. Further cohort and functional studies may help shedding a light on the related pathways altered at the organism level in prevalent PD, like red cells variability, inflammatory and oxidative stress mechanisms.

## 1 Introduction

The increased life expectancy achieved over the past three decades has led to an increase in the prevalence and burden of age-related neurodegenerative diseases (1). Among these, neurodegenerative disorders due to the accumulation of neurotoxic protein aggregates – prominently Alzheimer’s Disease (AD) and Parkinson’s Disease (PD) – represent the main concern from a public health perspective. Diagnostic and prognostic biomarkers are essential for early diagnosis and for predicting the course of these disorders. While some biomarkers have been proposed for AD risk prediction (2), in Parkinson’s disease reliable risk-predictive biomarkers are rare, making the search more urgent (3). In the last decades, powerful predictors of disease risk based on the use of epigenetic data have been developed, such as DNA methylation (*DNAm*, or epigenetic) age estimators, which represent useful markers of biological aging. These aging clocks reflect the discrepancy between the biological (i.e., actual) and the chronological age of an organism, with positive values suggesting an accelerated aging and negative values suggesting a decelerated aging. To date, biological aging clocks have been scarcely investigated for association with neurodegenerative disorders, in spite of their very good performance as age-related clinical risk predictors, reporting partly contrasting findings (see (4) for a comprehensive review). In spite of these promising findings, the evidence supporting a potential application of aging clocks in PD risk prediction is quite limited. Different case-control analyses reported PD patients to have a higher DNAm age based on different epigenetic clocks – including Hannum, Horvath, DNAm Phenotypic Age (DNAm PhenoAge) and epigenetic mitotic age (epiTOC) (5–7). Interestingly, some of these clocks were also associated with a faster cognitive decline and motor symptoms progression within patients (6). However, this association was not replicated in a longitudinal study, where only a strong positive association between Horvath’s DNAm age and an increased incident risk of PD was observed, along with an inverse association with age-at-onset (8). More recently, a two-sample Mendelian Randomization analysis reported DNAm GrimAge acceleration – a second generation methylation clock like PhenoAge (9) - to be marginally associated with an increased PD risk, although not significantly across all the models tested (10).

Overall, evidence reported so far supports an association of biological aging with both incident and prevalent risk of PD, with partly discordant results. Moreover, while interesting aging clocks based on blood markers have been proposed in the last years to estimate mortality and hospitalization risk in populations settings e.g (11–13), to our knowledge these were never tested for specific association with PD risk. Testing such aging clocks as potential predictors of incident PD may provide novel cost-effective tools for assessing neurodegenerative risk at the population level, and looks promising in view of previous associations of PD with several markers commonly assessed in blood tests, such as platelet parameters (peculiarly mean platelet volume and platelet distribution width) (14), and inflammatory markers like C-reactive protein levels (15) and neutrophil-to-lymphocyte ratio (16).

To clarify the relationship between systemic biological aging and Parkinson’s disease, here we tested for association a common aging clock based on diverse blood markers (PhenoAge (7)), with both incident and prevalent PD risk in a large Italian population cohort.

## 2 Subjects and methods

### 2.1 Population under study

The Moli-sani study is an ongoing prospective population-based cohort of 24,325 individuals (51.9% women, aged ≥ 35 years, mean age ± standard deviation (SD): 55.8 ± 12.0 years) living in the Molise region, in Southern-central Italy. Participants were randomly extracted from residents’ registries in several towns across the region and recruited between 2005 and 2010. Upon recruitment, each participant underwent both instrumental (e.g. blood tests, spirometry, electrocardiography) and questionnaire-based assessment, testing nutrition habits and other lifestyles, health status, history of disease, psychometric profiles and socioeconomic status (17), and since then was followed-up both actively and passively (see below). The study was approved by the Ethical Committee of the Catholic University of Rome and all the participants to the study provided written informed consent.

213 incident PD cases, whether living or deceased at the time of data capture, were identified until December 31, 2018, through individual-level record linkage with Molise regional registries of hospital discharge records (HDRs), the Italian registry of deaths (ReNCaM registry; “Registro Nominativo delle Cause di Morte”), and the regional drug prescription registry. Out of these incident cases, a further validation through cross-linking with IRCCS Neuromed internal database was feasible, which confirmed a PD clinical diagnosis and hence our correct classification in 100% of these cases. Further details on incident cases validation are reported in (18). Additional 52 prevalent PD cases were identified based on self-report of participants upon baseline recruitment.

### 2.2 Statistical analyses

All statistical analyses were performed in R v 4.0.4 (https://www.r-project.org/). Unreliable blood markers levels—i.e. leukocyte counts whose fractions summed < 99% (86) or > 101% (9)—were set to missing and missing values were then imputed through the k-nearest neighbor algorithm of the VIM package (19) (k = 10), as previously described in (13). After filtering out participants reporting non-Italian ancestry (332) and/or non-faster status at the time of blood draw (135), we computed Phenotypic Age (PhenoAge) in 23,858 participants, using the BioAge package (https://github.com/dayoonkwon/BioAge). PhenoAge is conceived as a blood-based biological age measure based on the application of Gompertz survival models to age and nine different circulating biomarkers, including albumin, creatinine, glucose, c-reactive protein, mean cell volume (MCV), red cell distribution width (RDW), alkaline phosphatase (ALP), white blood cell count (WBC) and lymphocyte fraction (7). Due to the lack of ALP within the Moli-sani cohort, a PhenoAge measure not including this marker was used, which was already reported to have comparable performance in mortality risk prediction and high correlation with the classical PhenoAge measure (13). Details on the markers available are reported in **Supplementary Table S1**. The discrepancy between chronological age and PhenoAge (hereafter called ΔPhenoAge) was computed for each participant and tested as an index of biological aging. This measure was reported to outperform deep learning markers of biological aging developed within the Moli-sani cohort (13) and was preferred over other (e.g. epigenetic) aging clocks due to the large availability of blood marker measures in the study.

Once computed, ΔPhenoAge was tested for association with both incident (testing biological aging as exposure) and prevalent PD risk (testing biological aging as outcome), to clarify the direction of the relationship between ΔPhenoAge and PD. For the former scope, starting from 23,858 subjects with a ΔPhenoAge measure available, we further removed (52) participants with self-reported PD diagnosis and (369) participants with missing follow-up information on incident PD. This left 23,437 observations (209 incident PD cases) for survival analysis. This analysis was carried out through multivariable Cox proportional hazards (PH) regressions to identify linear associations with incident PD risk, in incremental models adjusted for (i) age, sex (Model 1); (ii) Model 1 + education level completed (Model 2); (iii) Model 2 + other risk/protective factors (Model 3). These factors – including prevalent dysthyroidism, hypertension, type 2 diabetes, use of oral contraceptives, exposure to paints, daily coffee intake and cigarette smoking status - had been previously associated with incident PD risk in the same cohort (18). All the covariates used are explained in detail in Supplementary Methods and elsewhere (18). To model associations with prevalent PD risk, we carried out both and unpaired t-test of unadjusted ΔPhenoAge and a generalized linear model (Model 3) of ΔPhenoAge vs prevalent PD (yes/no). Statistical significance threshold was set to α = 0.05 and associations were deemed significant only if they were stable (p<0.05) in fully adjusted (glm) and unadjusted models (unpaired t-test). If significant associations were detected, further analyses of each component biomarker of PhenoAge available in the Moli-sani cohort were carried out, using the same tests and criteria mentioned above.

## 3 Results

Sociodemographic and clinical characteristics of the population under study are reported in **Table 1**. Compared to participants removed from the analysis, the analyzed sample showed a higher frequency of men (48.3% vs 42.7%, p = 0.001), of prevalent hypertension (57.5% vs 53.3%, p < 0.001) and a higher coffee intake (mean (SD): 82.0 (54.5) vs 75.0 (53.3) g/day, p < 0.001). Moreover, the analyzed samples showed on average a later detectable onset of PD in incident cases (74.9 (8.6) vs 64.9 (12.4) years, p = 0.006), although caution is suggested in the interpretation of these statistics given the low sample sizes involved (209 vs 4 patients; see **Table 1**).

**Table 1:** Baseline characteristics of the population under study.

Variable	Population under study (N = 23,437)	Removed participants (N = 888)	P for difference (analyzed vs removed)
Sex (men, %)	48.3	42.7	0.001
Age (y)	55.8 (11.9)	55.7 (13.1)	0.84
***Incident PD cases*** Mean (SD) age (y) at detected onset^a^ Min-max age (y) at detected onset^a^	209 74.9 (8.6) 47.9-95.0	4 64.9 (12.4) 39.5-83.4	1.00 0.006 NA
*Education (%)*			
Primary or less Lower secondary Upper secondary Post-secondary	25.7 27.8 34.1 12.4	29.2 27.1 31.5 12.2	0.13
Health conditions (%)			
Cancer Diabetes Pre-/Hypertension Dysthyroidism	3.5 5.0 29.4/57.5 6.4	3.2 6.1 29.3/53.3 6.3	0.71 0.14 <0.001 1.00
Lifestyles and other factors			
Current/previous smokers (%) Coffee intake (g/day) Exposure to paints (%) Use of oral contraceptives (current/past, %)	22.9/27.5 82.0 (54.5) 3.0 3.8/10.4	24.0/25.2 75.0 (53.3) 2.8 5.3/11.0	0.31 <0.001 0.92 0.05

Here we report frequency (%) for categorical variables, or, alternatively, mean values and standard deviations (SD) for continuous variables. The population under study results from the exclusion of participants, with lack of follow-up information, PhenoAge or sociodemographic covariates required for basic Cox PH models (Model 1), or with a prevalent PD condition. P-values resulting from statistical comparisons of the analyzed vs non-analyzed participants are reported, rounded to the second decimal place, unless they were significant. Chi-squared test was applied to education level, hypertension, smoking status and use of oral contraceptives; Fisher Exact Test to sex, prevalent cancer, diabetes and dysthyroidism; unpaired t-test to age and daily coffee intake; Mann-Whitney U Test to age at detected PD onset. All the variables are defined in **Supplementary Materials**.

**Figure 1:**
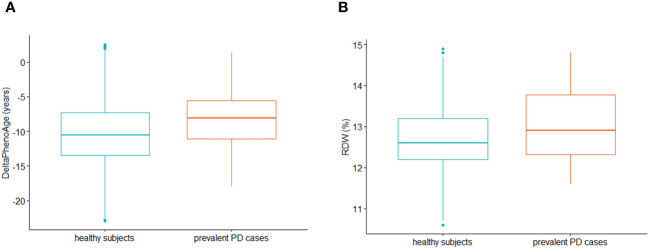
These boxplots compare **(A)** ∆PhenoAge (i.e. the difference between PhenoAge and chronological age) and **(B)** red cell distribution width (RDW) distributions between subjects not reporting (green, N=23,385; median (IQR) ∆PhenoAge: -10.6 (6.2) years; median (IQR) RDW: 12.6 (1.0)%) and those reporting prevalent PD at baseline recruitment (red, N=52; ∆PhenoAge: -8.1 (5.5) years; RDW: 12.9 (1.5)%). The distributions were plotted after removal of outliers in the total population under study, based on Interquartile Range (IQR) rule (i.e., all the observations with ∆PhenoAge above/below mean ∆PhenoAge ± 1.5*IQR in the whole analyzed sample were removed). Dots represent single outliers which were observed within each compared group, based on the IQR rule (note that they may go undetected in the analysis of the whole sample due to different distributions).

Multivariable Cox PH regressions modelling the risk of incident PD vs ΔPhenoAge (209 incident cases, median (IQR) follow-up time 11.19 (2.03) years) revealed no significant associations with incident PD cases (Model 1: Hazard Ratio [95% Confidence Interval] = 1.00 [0.98; 1.02]); Model 2: 1.00 [0.98; 1.02]; Model 3: 0.98 [0.71; 1.37]). When modelling prevalent PD status as exposure and ΔPhenoAge as outcome, a t-test for independent samples revealed a significant discrepancy between subjects reporting and those not reporting prevalent PD status, the former being biologically older than the latter (mean (SD) unadjusted ΔPhenoAge: -7.32 (6.27) vs -9.83 (5.36) years, 95% CI of difference: [-4.26; -0.77], t = -2.89, p = 0.006; **Figure 1A**). Similarly, generalized linear regression models revealed a modest but significant increase of ΔPhenoAge in prevalent PD cases vs healthy subjects, which was stable across incrementally adjusted models (Model 1: β (Standard Error) = 1.49 (0.73) years; Model 2: 1.47 (0.73); Model 3: 1.39 (0.70), p = 0.047). A further analysis of each component biomarker of PhenoAge available in the cohort revealed a significant positive association of prevalent PD status with RDW, both in a pairwise comparison between participants reporting vs not reporting a PD diagnosis (13.51 (1.67) vs 12.93 (1.22)%, 95% CI of difference: [0.10; 1.08]; **Figure 1B**), and in fully adjusted regression models (**Table 2**). All the remaining markers did not show any robust evidence of association.

**Table 2:** PhenoAge component biomarkers vs prevalent PD status.

PhenoAge component biomarker (unit)	Glm β (SE), p-value	Prevalent PD: Mean (SD)	Healthy subjects: Mean (SD)	Unpaired samples t-test (p)	Difference [95% CI]
**WBC (×10^3^/µl)**	-0.11 (0.24), 0.66	6.12 (1.45)	6.23 (1.78)	t = -0.54 (p = 0.59)	[-0.52; 0.30]
**LY (%)**	-1.84 (1.06), 0.08	30.40 (6.54)	32.65 (7.34)	t = -2.38 (p = 0.02)	[-4.14; -0.35]
**CRP (mg/L, log-scale)**	-0.045 (0.14), 0.74	0.58 (0.98)	0.42 (1.03)	t = 1.14 (p = 0.26)	[-0.12; 0.43]
**Creatinine (mg/dL)**	0.03 (0.03), 0.23	0.88 (0.29)	0.82 (0.21)	t = 0.00 (p = 1.00)	[-0.003; 0.004]
**Albumin (g/dL)**	-0.04 (0.06), 0.46	4.11 (0.25)	4.21 (0.32)	t = -2.17 (p = 0.04)	[-0.18; -0.01]
**Glucose (mg/dL)**	-1.41 (2.91), 0.63	109.38 (31.28)	101.52 (25.40)	t = 1.81 (p = 0.08)	[-0.85; 16.58]
**MCV (fL)**	-0.52 (0.83), 0.53	89.07 (6.63)	88.34 (6.04)	t = 0.78 (p = 0.44)	[-1.16; 2.61]
**RDW (%)**	**0.46 (0.18),** **0.009**	**13.51 (1.67)**	**12.93 (1.22)**	**t = 2.41 (p = 0.02)**	**[0.10; 1.08]**

Here the results (unstandardized β and Standard Error) of fully adjusted generalized linear models (glm, Model 3) of each component biomarker of PhenoAge vs prevalent PD status are reported, along with the results of t-test for unpaired samples and the 95% Confidence Interval of the difference between means of participants reporting vs not reporting PD diagnosis. Markers showing significant associations with ∆PhenoAge both in the fully adjusted glm and in the unpaired t-test (p<0.05) are highlighted in bold. WBC, white blood cell count; LY, lymphocyte fraction over WBC; CRP, c-reactive protein, MCV, mean cell volume; RDW, red cell distribution width.

## 4 Discussion

In the present manuscript, we investigated the relationship between a systemic marker of biological aging (specifically phenotypic aging, or ΔPhenoAge), and both the incident and the prevalent risk of Parkinson’s Disease in an Italian population cohort. We observed a small but significant increase in phenotypic aging of participants reporting a previous diagnosis of PD, compared to those not reporting it, but found no evidence of association between ΔPhenoAge and incident PD risk. While DNAm PhenoAge has been previously associated with all-cause/cause-specific mortality and with other age-related chronic disorders such as Alzheimer’s disease (7) and cancer (20), this finding is not surprising considering the few observations reported so far for Parkinson’s disease. Indeed, some case-control studies found concordant evidence with our work, indicating PD patients to have a higher DNAm age based on different epigenetic clocks (5–7). Interestingly, these studies tested different types of blood-based epigenetic markers – including Hannum and Horvath clocks (5), DNAm PhenoAge (7) and epiTOC acceleration (6) – in large case-controls studies (with number of cases in the range [289-569]). A recent bidirectional Mendelian Randomization analysis based on summary statistics of large Genome Wide Association Studies (GWAS) supported this evidence, suggesting a causative link between PD status (modelled as exposure) and DNAm GrimAge acceleration (outcome), among different aging clocks tested (10). Of interest, the association was not supported when modelling DNAm age acceleration as exposure and PD status as outcome.

However, a longitudinal analysis of two cohorts of idiopathic PD patients and a cohort of *LRRK2* G2019S mutation carriers identified a strong influence of Horvath clock acceleration on incident PD risk, revealing that a 5-year increase in DNAm age acceleration was associated with a 6-year earlier PD onset (8), in contrast with the evidence reported here and elsewhere (10). While it would be tempting to explain this discrepancy through a potential influence that biological aging may have in subjects at high genetic risk of PD, the available evidence it too scarce to make speculations and further studies are warranted.

Among all the component biomarkers of PhenoAge acceleration tested in our cohort – only RDW showed a significant association with prevalent PD, being higher in subjects reporting prevalent PD, in line with previous evidence reported both for PD (21) and for other neurodegenerative disorders like AD (22). RDW also showed a significant correlation with disease severity (Unified Parkinson’s Disease Rating Scale, UPDRS) and staging (modified

Hoehn and Yahr score) (23), although not concordantly across studies (21).

RDW is considered a novel biomarker of oxidative stress and inflammation, which may result from the inhibited maturation of erythropoietin-induced erythrocytes or from a reduction of the red blood cells’ lifespan. Indeed, RDW correlates with inflammatory markers such as C-reactive protein (CRP), erythrocyte sedimentation rate and several inflammatory cytokines (21). This underlines the implication of systemic inflammatory pathways in PD (24), warranting further studies to identify the source of such alterations. Alternatively, the association observed here may be due to horizontal pleiotropy of specific genetic variants affecting both RDW and PD risk, as suggested by a previous GWAS study within the UK Biobank, reporting that some genetic variants associated with RDW variability had also been previously associated with PD (25).

### 4.1 Strengths and limitations

Our study presents elements of novelty since for the first time we tested a blood-based (non-epigenetic) biological aging clock for association with both incident and prevalent PD risk in the same study, in a large population cohort. Other strengths include the length of follow-up and the wealth of covariates available for adjustment. However, the present work also suffers from some limitations. First, we could not compute PhenoAge as described in the original manuscript (7) due to the lack of ALP, but used a proxy measure which we already proved to be highly correlated with it and to have comparable risk-predictive performance in the Moli-sani cohort (13). Second, the number of both incident and – most of all - prevalent PD cases in our cohort is relatively low compared to other studies (<300), but this is somehow unavoidable when facing a general population cohort and is expected to improve with increasing follow-up duration. Also, this cohort may not be representative of the Italian population, although PD incidence and prevalence are in line with other Italian regions (18). Third, currently we have no information on age at onset and clinical characteristics of prevalent PD cases - which may help testing further relationships with disease endophenotypes - but are working on improving this aspect through cross-validation with clinical records, where feasible. Fourth, the lack of genetic data did not allow us to determine whether the presence of rare mutations with strong effect size may somehow influence the relationship between PhenoAging and PD in some of the analyzed cases. However, the low frequency of early onset cases (only one participant with detectable onset <50 years) makes it unlikely that such association be confounded by familial PD forms under strong genetic influence. Fifth, we tested a blood-based clock, which may not represent the actual biological aging of central nervous system, that is primarily affected by PD. However, the evidence reported here may indicate novel pathways altered at the organism level in prevalent PD, like inflammation and oxidative stress, as suggested by the evidence related to RDW.

## 5 Conclusions

Overall, the available evidence makes it difficult to establish blood biomarkers as robust predictors of incident neurodegenerative risk in the general population - warranting further studies in larger longitudinal cohorts - but highlights systemic effects of prevalent PD status on biological aging and red cell distribution width. These results pave the way for new hypotheses on the pathophysiology of this neurodegenerative disorder, implicating red cells variability, as well as potentially related inflammatory and oxidative stress mechanisms. Further cohort and functional studies may help shedding a light on the specific pathways altered at the organismal level in prevalent PD.

## Data availability statement

The dataset presented in this study can be found in online repository https://repository.neuromed.it. The password for accessing raw data will be provided upon reasonable request to the corresponding author.

## Ethics statement

The studies involving humans were approved by Ethical Committee of the Catholic University of Rome. The studies were conducted in accordance with the local legislation and institutional requirements. The participants provided their written informed consent to participate in this study.

## Author contributions

AG: Conceptualization, Formal analysis, Investigation, Methodology, Supervision, Writing – original draft. AT: Formal analysis, Investigation, Visualization, Writing – original draft. SC: Data curation, Investigation, Validation, Writing – review & editing. MIDB: Data curation, Resources, Validation, Writing – review & editing. DB: Data curation, Resources, Validation, Writing – review & editing. SM: Data curation, Investigation, Writing – review & editing. ADC: Data curation, Investigation, Writing – review & editing. SF: Resources, Writing – review & editing. MR: Resources, Writing – review & editing. CC: Conceptualization, Project administration, Resources, Writing – review & editing. MBD: Conceptualization, Funding acquisition, Writing – review & editing. AB: Data curation, Resources, Validation, Writing – review & editing. GdG: Conceptualization, Funding acquisition, Project administration, Writing – review & editing. LI: Conceptualization, Funding acquisition, Project administration, Supervision, Writing – review & editing.

## Group members of Moli-sani Study Investigators

A complete list of the Moli-sani Study Investigators is available in **Supplementary Materials**.
