# Supplementary material for: Blood-based biological ageing and red cell distribution width are associated with prevalent Parkinson’s disease: findings from a large Italian population cohort
Source: Front Endocrinol (Lausanne). 2024 Apr 10;15:1376545. doi: 10.3389/fendo.2024.1376545 (PMC11041016; doi:10.3389/fendo.2024.1376545)
Supplement: Supplementary file 1 [file DataSheet_1.docx]

**Supplementary Methods**

***Circulating biomarkers***

Blood samples were obtained between 7 and 9 AM from participants who had fasted overnight and had refrained from smoking for at least 6 hours (h). Biochemical analyses were performed in the centralized Moli-sani laboratory, as described elsewhere [1]. Haemochromocytometric analyses were performed by cell counter (Coulter HMX, Beckman Coulter, Milan, Italy) within 3 h from venipuncture. Analyses of creatinine were performed within the BiomarCaRE Consortium and have been described in details elsewhere (see <https://cordis.europa.eu/project/id/278913/reporting/it>) [2]. Main information and descriptive statistics for all the circulating biomarkers currently available in the Moli-sani study is reported in Table S1.

***Definition of covariates***

Education level was used as a socioeconomic status proxy and classified as primary (reference class), lower, upper, or post-secondary. Smoking status of participants was classified as never-smokers (reference class), current smokers, or previous smokers (i.e. non-smoking for at least 1 year). Dietary intake was assessed by an interviewer-administered and semiquantitative questionnaire, the European Prospective Investigation into Cancer and Nutrition Food Frequency Questionnaire (EPIC-FFQ), which was validated and adapted for the Italian population to assess participants’ diets during the 12 months preceding recruitment [3, 4]. Coffee consumption was assessed with a question regarding the consumption frequency of caffeinated and decaffeinated coffee, the two types mainly consumed in Italy in the form of espresso or percolated coffee. The questionnaire asked “How many cups of coffee do you usually drink?”, with possible answers being number of cups per day, week, or month, or never/almost never. Subsequently, participants were asked to indicate the type of coffee usually consumed, with options including Italian espresso (30 mL), mocha coffee (50 mL), or decaffeinated coffee (30 mL). Cappuccino and café latte were assumed to contain 20% coffee and 80% milk. We then calculated total coffee consumption (g/day; 1 mL of coffee = 1 g) based on the total consumption of all coffee drinks [5]. Information on self-reported current or past occupational exposure to paints was also collected. Systolic (SBP) and diastolic blood pressure (DBP) was measured by an automatic device (OMRON-HEM-705CP) as described elsewhere [6]. Pre-hypertension was defined as DBP in the range [80-90) mmHg and SBP in the range [120-140) mmHg, while hypertension was defined as blood pressure ≥140/90 mmHg or use of specific pharmacological treatments.

Moli-sani study participants were also asked to provide accurate information on the use (frequency and dose) of medication taken to treat any disease they suffered and the questionnaire on drug usage was directly linked to the Italian national drug index. This way, we were able to detect for each participant the use of hypoglycemic drugs (used as a proxy of type 2 diabetes status). Similarly, we were able to assess the current and past use of oral contraceptives, which was compared to never users (reference class). Dysthyroidism was defined based on the self-reported use of drugs for thyroid therapy and classified as a binary variable, as was self-reported history of cancer. Further details on these variables can be found in [7].

**Table S1. Details and main descriptive statistics of all the blood markers used to compute PhenoAge in the Moli-sani cohort.**

| Variable | Source | Unit | Observations | # Missing | Missing (%) | Min | Max | Median | Mean | SD |
| --- | --- | --- | --- | --- | --- | --- | --- | --- | --- | --- |
| WBC | blood | ×10^3^/µl | 23666 | 659 | 2.7 | 1.40 | 68.80 | 6.00 | 6.23 | 1.78 |
| LY | blood | % | 23450 | 875 | 3.6 | 6.30 | 90.20 | 32.40 | 32.64 | 7.33 |
| MCV | blood | fl | 23665 | 660 | 2.7 | 10.20 | 123.90 | 89.00 | 88.33 | 6.04 |
| RDW | blood | % | 23544 | 781 | 3.2 | 0.27 | 33.90 | 12.70 | 12.93 | 1.23 |
| Glucose | serum | mg/dl | 24174 | 151 | 0.6 | 12.00 | 443.00 | 97.00 | 101.49 | 25.40 |
| CRP | serum | mg/l | 24297 | 28 | 0.1 | 0.01 | 20.00 | 1.52 | 2.59 | 3.26 |
| Albumin | serum | g/dl | 16270 | 8055 | 33.1 | 1.80 | 5.60 | 4.20 | 4.21 | 0.32 |
| Creatinine | serum | mg/dl | 23647 | 678 | 2.8 | 0.33 | 7.13 | 0.78 | 0.82 | 0.21 |

Abbreviations: WBC = total white blood cell count; LY = lymphocyte fraction over WBC; MCV = Mean Corpuscular Volume; RDW = red cell distribution width; CRP = high sensitivity C-reactive protein.

**Figure S1.** Cox survival curves of incident PD risk vs Phenotypic Aging.


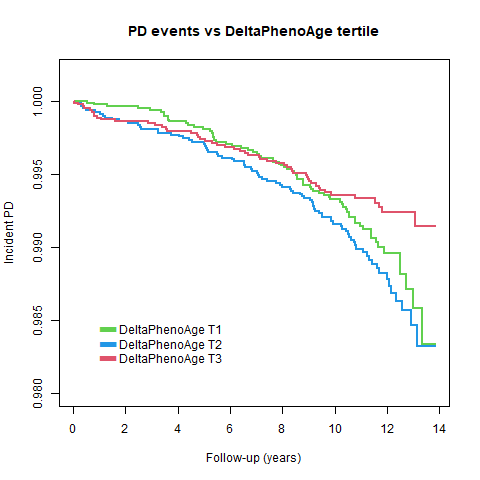


For this plot, ∆PhenoAge was first residualized against all the covariates used in the fully adjusted model (Model 3, see Methods section), then divided into tertiles (T1: ∆PhenoAge in [-37.01; -2.24]; T2: (-2.24; 1.38]; T3: (1.38; 59.57]), which were compared in terms of incident PD rate during follow-up.

**Figure S2.** Scatter plot of Phenotypic Aging vs PD status.


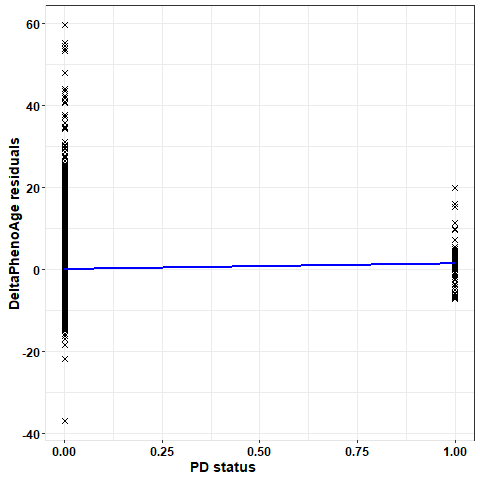


Each cross represents a subject, while interpolation line is highlighted in blue. For this plot, ∆PhenoAge was first residualized against all the covariates used in the fully adjusted model (Model 3, see Methods section), then plotted separately for prevalent PD cases (PD status = 1) and healthy subjects (PD status = 0).

**References**

1. Santimone, I., et al., *White blood cell count, sex and age are major determinants of heterogeneity of platelet indices in an adult general population: results from the MOLI-SANI project.* Haematologica, 2011. **96**(8): p. 1180-8.

2. Zeller, T., et al., *BiomarCaRE: rationale and design of the European BiomarCaRE project including 300,000 participants from 13 European countries.* European Journal of Epidemiology, 2014. **29**(10): p. 777-790.

3. Pisani, P., et al., *Relative validity and reproducibility of a food frequency dietary questionnaire for use in the Italian EPIC centres.* Int J Epidemiol, 1997. **26 Suppl 1**: p. S152-60.

4. Pala, V., et al., *Diet in the Italian EPIC cohorts: presentation of data and methodological issues.* Tumori, 2003. **89**(6): p. 594-607.

5. Ruggiero, E., et al., *Daily Coffee Drinking Is Associated with Lower Risks of Cardiovascular and Total Mortality in a General Italian Population: Results from the Moli-sani Study.* J Nutr, 2021. **151**(2): p. 395-404.

6. Bonaccio, M., et al., *Nutrition knowledge is associated with higher adherence to Mediterranean diet and lower prevalence of obesity. Results from the Moli-sani study.* Appetite, 2013. **68**: p. 139-46.

7. Gialluisi, A., et al., *Risk and protective factors in Parkinson's disease: a simultaneous and prospective study with classical statistical and novel machine learning models.* J Neurol, 2023. **270**(9): p. 4487-4497.

**Moli-sani Study Investigators**

The enrolment phase of the Moli-sani Study was conducted at the Research Laboratories of the Catholic University in Campobasso (Italy), the follow up of the Moli-sani cohort is being conducted at the Department of Epidemiology and Prevention of the IRCCS Neuromed, Pozzilli, Italy.

**Steering Committee:** Licia Iacoviello*^#^(Chairperson), Giovanni de Gaetano*, Maria Benedetta Donati*.

**Scientific Secretariat:** Chiara Cerletti* (Coordinator), Marialaura Bonaccio*, Americo Bonanni*, Simona Costanzo*, Amalia De Curtis*, Augusto Di Castelnuovo^§^, Alessandro Gialluisi*^#^, Francesco Gianfagna^§^°, Mariarosaria Persichillo*, Teresa Di Prospero* (Secretary).

**Safety and Ethical Committee:** Jos Vermylen (Catholic University, Leuven, Belgio) (Chairperson), Renzo Pegoraro (Pontificia Accademia per la Vita, Roma, Italy), Antonio Spagnolo (Catholic University, Roma, Italy).

**External Event Adjudicating Committee**: Deodato Assanelli (Brescia, Italy), Livia Rago (Campobasso, Italy).

**Baseline and Follow-up Data Management:** Simona Costanzo* (Coordinator), Marco Olivieri (Campobasso, Italy), Sabatino Orlandi*, Teresa Panzera*.

**Data Analysis:** Augusto Di Castelnuovo^§^ (Coordinator), Marialaura Bonaccio*, Simona Costanzo*, Simona Esposito*, Alessandro Gialluisi*^#^, Anwal Ghulam*, Francesco Gianfagna^§^°, Roberta Parisi, Antonietta Pepe*, Emilia Ruggiero*, Sukshma Sharma*.

**Biobank, Molecular and Genetic Laboratory:** Amalia De Curtis* (Coordinator), Concetta Civitillo*, Alisia Cretella*, Sara Magnacca^§^, Fabrizia Noro*.

**Recruitment Staff:** Mariarosaria Persichillo* (Coordinator), Francesca Bracone*, Giuseppe Di Costanzo*, Sabrina Franciosa*, Martina Morelli*, Teresa Panzera*.

**Communication and Press Office:** Americo Bonanni*.

**Regional Institutions:** Direzione Generale per la Salute - Regione Molise; Azienda Sanitaria Regionale del Molise (ASReM, Italy); Agenzia Regionale per la Protezione Ambientale del Molise (ARPA Molise, Italy); Molise Dati Spa (Campobasso, Italy); Offices of vital statistics of the Molise region.

**Hospitals:** Presidi Ospedalieri ASReM: Ospedale A. Cardarelli – Campobasso, Ospedale F. Veneziale – Isernia, Ospedale San Timoteo - Termoli (CB), Ospedale Ss. Rosario - Venafro (IS), Ospedale Vietri – Larino (CB), Ospedale San Francesco Caracciolo - Agnone (IS); Casa di Cura Villa Maria - Campobasso; Responsible Research Hospital - Campobasso; IRCCS Neuromed - Pozzilli (IS).

***Department of Epidemiology and Prevention, IRCCS Neuromed, Pozzilli, Italy

^#^Department of Medicine and Surgery, LUM University “Giuseppe Degennaro”, Casamassima, Italy

^§^Mediterranea Cardiocentro, Napoli, Italy

°Department of Medicine and Surgery, University of Insubria, Varese, Italy

*Moli-sani Study Past Investigators are available at* [*https://www.moli-sani.org/?page_id=173*](https://www.moli-sani.org/?page_id=173)
